# Regulation of autophagy and lipid accumulation under phosphate limitation in *Rhodotorula toruloides*

**DOI:** 10.3389/fmicb.2022.1046114

**Published:** 2023-01-26

**Authors:** Ya-nan Wang, Fang-jie Liu, Hong-di Liu, Yue Zhang, Xiang Jiao, Ming-liang Ye, Zong-bao Kent Zhao, Su-fang Zhang

**Affiliations:** ^1^Laboratory of Biotechnology, Dalian Institute of Chemical Physics, CAS, Dalian, China; ^2^State Key Laboratory Breeding Base of Dao-di Herbs, National Resource Center for Chinese Materia Medica, China Academy of Chinese Medical Sciences, Beijing, China; ^3^Key Laboratory of Separation Sciences for Analytical Chemistry, Dalian Institute of Chemical Physics, CAS, Dalian, China; ^4^Dalian Key Laboratory of Energy Biotechnology, Dalian Institute of Chemical Physics, CAS, Dalian, China

**Keywords:** *Rhodotorula toruloides*, phosphate limitation, autophagy, Atg9, lipid accumulation

## Abstract

**Background:**

It is known that autophagy is essential for cell survival under stress conditions. Inorganic phosphate (Pi) is an essential nutrient for cell growth and Pi-limitation can trigger autophagy and lipid accumulation in oleaginous yeasts, yet protein (de)-phosphorylation and related signaling events in response to Pi limitation and the molecular basis linking Pi-limitation to autophagy and lipid accumulation remain elusive.

**Results:**

Here, we compared the proteome and phosphoproteome of *Rhodotorula toruloides* CGMCC 2.1389 under Pi-limitation and Pi-repletion. In total, proteome analysis identified 3,556 proteins and the phosphoproteome analysis identified 1,649 phosphoproteins contained 5,659 phosphosites including 4,499 pSer, 978 pThr, and 182 pTyr. We found Pi-starvation-induced autophagy was regulated by autophagy-related proteins, but not the *PHO* pathway. When *ATG*9 was knocked down, the engineered strains produced significantly less lipids under Pi-limitation, suggesting that autophagy required Atg9 in *R*. *toruloides* and that was conducive to lipid accumulation.

**Conclusion:**

Our results provide new insights into autophagy regulation under Pi-limitation and lipid accumulation in oleaginous yeast, which should be valuable to guide further mechanistic study of oleaginicity and genetic engineering for advanced lipid producing cell factory.

## Introduction

The red yeast *Rhodotorula toruloides* is a basidiomycetous yeast that has been explored for the production of lipids, oleochemicals and related products (Hu et al., 2009; Xu et al., 2012; Huang et al., 2013; Wen et al., 2020). While lipid production by oleaginous yeasts occurs often under nitrogen limitation, phosphate (Pi) limitation has also been demonstrated effective to promote lipid accumulation (Wu et al., 2010; Wang et al., 2018). Since phosphorus is an essential element, *R*. *toruloides* cells must acquire phosphorus from the external environment during proliferation under Pi-limitation. One of the key cellular responses to nutrient depletion is the upregulation of autophagy.

Autophagy is an important cellular process that enables the delivery of cytoplasmic contents and organelles to the vacuole or lysosome for recycling or degradation (Levine and Klionsky, 2004; Nakatogawa et al., 2009); it can be induced by depletion of various nutrients such as nitrogen, amino acids, carbon, sulfur, zinc, and Pi in yeasts (Takeshige et al., 1992; Kamada et al., 2000; Kawamata et al., 2017; Yokota et al., 2017). The TORC1 signaling pathway senses nutrients and negatively regulates autophagy. In budding yeast, when nutrients are deficient, the target of rapamycin complex 1 (TORC1) activity is inhibited. The conserved TOR complex is present in *R*. *toruloides* and nitrogen limitation inactivates TORC1; inhibits ribosome biogenesis and activates autophagy (Zhu et al., 2012). Pi-starvation-induced autophagy (PSiA) is nonselective autophagy. Interestingly, Atg11, an adaptor protein for selective autophagy, proved to achieve bulk autophagy in Pi starvation conditions in *Saccharomyces cerevisiae* (Yokota et al., 2017). However, the regulation of autophagy under Pi limitation has not been studied in oleaginous yeast *R*. *toruloides*. Previously, a number of post-translational modifications (PTM) on various Atg proteins have been studied and showed to be crucial in regulating autophagy activity. Moreover, protein phosphorylation is the best-characterized PTM on Atg proteins (Cherra et al., 2010; Yi et al., 2012; Papinski and Kraft, 2014). In eukaryotes, phosphorylation occurs more frequently on serine (Ser) and threonine (Thr) than tyrosine (Tyr; Yuan et al., 2016). Nitrogen starvation enables a dephosphorylated Atg13 to interact with Atg1 to form an active autophagy initiation complex. However, Pi starvation caused Atg13 dephosphorylation with slower kinetics than nitrogen starvation, and induced poor autophagic activity (Yokota et al., 2017). Furthermore, the multiple spanning integral membrane protein Atg9 phosphorylation is required for the efficient recruitment of Atg8 and Atg18 to the site of autophagosome formation (Papinski et al., 2014); making it an essential component of the autophagic molecular machinery.

Currently, the phosphorylation targets and signaling mechanism for autophagy under Pi limitation remains not well understood. Because phosphorylated proteins, by nature, are reversible and ubiquitous, readily converted from one form to the other. In a typical large-scale phosphorylation analysis, a preliminary enrichment step of phosphopeptides is essential to reduce sample complexity and increase their relative concentration. A wide variety of phosphopeptide enrichment strategies have been proposed, including chemical approaches using *β*-elimination or phosphoramidate chemistry (Oda et al., 2001; Zhou et al., 2001; Tao et al., 2005), peptide immunoprecipitation with phospho-specific motif antibodies (Rush et al., 2005), affinity purification through metal complexation with the Pi group [immobilized metal ion affinity chromatography (IMAC; Ficarro et al., 2002)], acid base interaction with TiO_2_ (Larsen et al., 2005), solution-charge-based enrichment by strong cation exchange (SCX) chromatography (Beausoleil et al., 2004), and combinations of these (Gruhler et al., 2005; Zhang et al., 2005).

The aim of this study was to gain insight into the regulation of autophagy in *R*. *toruloides* in response to Pi limitation. In addition, although there are many studies on protein phosphorylation, little is known about the phosphorylation of proteins under Pi limitation. In this work, we compared and analyzed the proteome and phosphoproteome of *R*. *toruloides* strains under Pi limitation and Pi replete condition.

## Materials and methods

**Figure 1:**
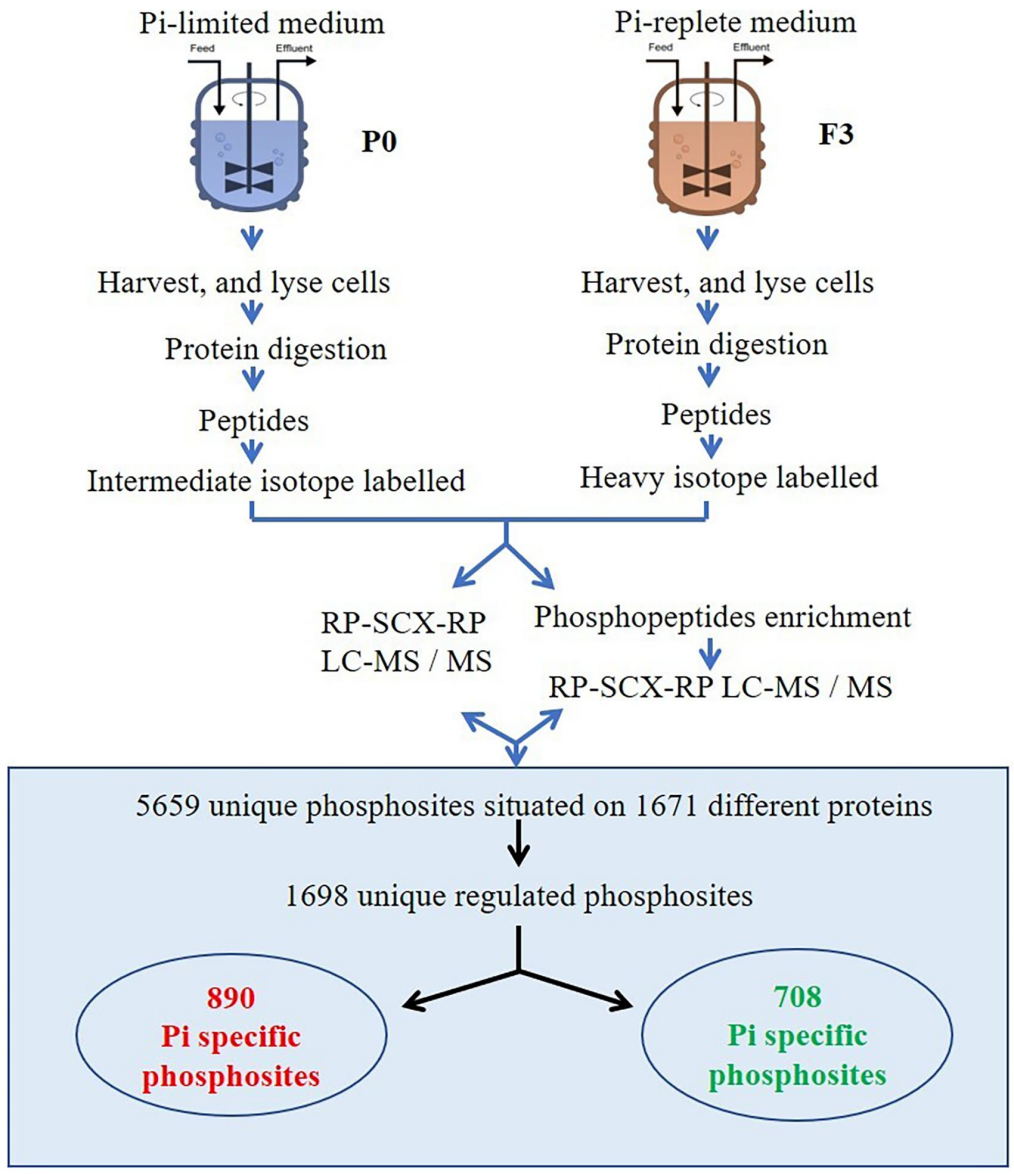
Overview of the experimental and analytical strategy.

Overview of the experimental and analytical strategy is shown in Figure 1.

### Chemostat cultivations: Strains and media

The red yeast *R*. *toruloides* CGMCC 2.1389 (China General Microbiological Culture Collection Center) was grown in 3-l stirred tank reactor (Baoxing Bio-Engineering Equipment, Shanghai, China) with a constant working volume of 2.0 liter. The bioreactor was equipped with a 405-DPAS-SC-K8S/225 pH electrode and an InPro 6,800 O_2_ sensor (Mettler Toledo).

The seed medium contained (g l^−1^): glucose·H_2_O 30, (NH_4_)_2_SO_4_ 5.0, Na_2_HPO_4_ 1.0, KH_2_PO_4_ 1.0, and MgSO_4_ 1.5, pH 6.0. The Pi-limited (PL) lipid production medium contained (g l^−1^): glucose·H_2_O 30, (NH_4_)_2_SO_4_ 5.0, Na_2_SO_4_ 0.94, Na_2_HPO_4_ 0.06, K_2_SO_4_ 0.64, and MgSO_4_ 1.5, pH 6.0 with a C/P molar ratio of 2,164 and the Pi-replete (PR) medium (g l^−1^): glucose·H_2_O 30, (NH_4_)_2_SO_4_ 5.0, Na_2_HPO_4_ 1.0, KH_2_PO_4_ 1.0, and MgSO_4_ 1.5, pH 6.0 with a C/P molar ratio of 63. After being sterilized at 121°C for 15 min, the medium was supplemented with a 100-fold diluted trace element solution (Li et al., 2007). The trace element solution contained per liter: CaCl_2_·2H_2_O 4.0 g, FeSO_4_·7H_2_O 0.55 g, ZnSO_4_·7H_2_O 0.10 g, MnSO_4_·H_2_O 0.076 g, and 100 μL of 18 M H_2_SO_4_, and was sterilized at 121°C for 15 min.

The seed culture was done at 30°C in 250-mL Erlenmeyer flask on a rotary shaker at 200 rpm for 28 h. The chemostat culture was initiated by adding 200 mL of the seed culture into 1.8-L of sterilized lipid production medium and held at 30°C, pH of 5.6 (maintained by automatic addition of 2 M NaOH) with a stirrer speed of 500 rpm and a dissolved oxygen above 85% of air saturation. The airflow was 100 l h^−1^. The dilution rate was 0.085 h^−1^ and 0.3 h^−1^ for the culture feeding with the PL medium and the PR medium, and the sample was designated as P0 (PL condition) and F3 (PR condition), respectively. Steady-state samples were taken after five volume changes.

### Quantitative proteomics and phosphoproteomics enabled By stable-isotopic dimethyl labeling

For cell lysis, cultured yeast cells were thoroughly ground in a mortar in the presence of liquid nitrogen. The homogenate was suspended in 1 × TEAB (Triethylammonium bicarbonate, Sigma-Aldrich, 100 mM, pH 7.4) buffer supplemented with 8 M Urea, 65 mM dithiothreitol (DTT), 1 mM EDTA, 0.5 mM EGTA, phosphatase inhibitor (1 mM NaF and 1 mM Na_3_VO_4_), 1% Triton X-100 (v/v) and 2% protease inhibitor cocktail (v/v, Sigma-Aldrich, United States) and then sonicated in ice bath at 400 W for 120 s. If the extracted protein was not used for phosphoproteome analysis, there was no need to add the phosphatase inhibitor into the lysis buffer. For proteomic analysis, the protein sample was obtained by methanol-chloroform treatment. Briefly, the cell lysate was centrifuged at 25,000 *g* at 4°C for 30 min. One volume of the supernatant was mixed with four volumes of methanol and vortexed for 30 s; then mixed with one volume of chloroform and vortexed for another 30 s, and last mixed with three volumes of water and vortexed for 30 s. The mixture was centrifuged at 14,000 *g* for 3 min at room temperature, and the upper aqueous phase was discarded while keeping the white precipitate (the protein precipitate is between the two layers). Another four volumes of methanol were added, and vortexed for 30 s followed by centrifugation at 14,000 *g* for 3 min. The supernatant was removed as much as possible without disturbing the protein pellets. Finally, the protein pellets were obtained for bottom-up proteomics.

The obtained proteins were dissolved in 8 M Urea, 100 mM TEAB buffer (pH 8.2), and the concentration were determined by Bradford reagent assay kit (Beyotime Biotechnology Corporatio4n, Shanghai, China). For protein digestion, the sample was reduced with 20 mM DTT at 37°C for 2 h and alkylated with 40 mM IAA for 40 min, respectively. The solution was diluted to 1 M urea using 100 mM TEAB buffer (pH 8.2) and incubated with trypsin at the ratio of trypsin-to-protein of 1/25 (w/w) at 37°C overnight. All the resulting peptides were stored at −80°C.

For quantitative proteomics enabled by stable-isotopic dimethyl labeling, the P0 and F3 samples were labeled with intermediate and heavy dimethyl isotope labels, respectively. Firstly, 500 μL of CD_2_O (4%, V/V) and ^13^CD_2_O (4%, V/V) were added into the P0 and F3 samples, respectively, and then 500 μL of freshly prepared NaBH_3_CN (0.6 M) and NaBD_3_CN (0.6 M) were added subsequently to both samples. The resultant mixtures was incubated for 1 h at room temperature followed by the addition of 20 μL of 25% ammonia (v/v) and stand for 15 min. 50 μL of FA was added to react with the excess labeling reagents to terminate the reaction. Finally, the two labeled samples were mixed in the ratio of 1:1 (w/w) based on the total peptide amount. The labeling peptide mixtures were desalted on an OASIS HLB column, lyophilized and stored at −80°C for further analysis.

### Phosphopeptides enrichment by Ti (IV)-immobilized metal ion affinity chromatography beads

The labeled peptides were enriched by Ti (IV)-IMAC microspheres without any desalting step. Briefly, the peptide solution was incubated with Ti (IV)-IMAC beads at the ratio of 1:10 (w/w) in the loading buffer (80% ACN, 6% TFA), and then the beads were washed by washing buffer 1 (50% ACN, 6% TFA, 200 mM NaCl) and washing buffer 2 (30% ACN, 0.1% TFA), respectively to remove the non-specifically bound peptides (Zhou et al., 2013). The bound phosphopeptides was finally eluted by 10% ammonia-water (v/v) and dried down in a Speed-Vac.

### Online reversed phase-strong cation exchange-reversed phase multidimensional separation and mass spectrometry analysis

Lyophilized peptides and phosphopeptides were redissolved in 0.1% FA/H_2_O for online RP-SCX-RP LC–MS/MS analysis. It was performed on LTQ Orbitrap Velos (Thermo Fisher Scientific, San Jose, CA, United States), with an Accela 600 HPLC system for separation (Thermo Fisher Scientific, San Jose, CA, United States). The automatic sample injection and multidimensional separation system included a RP-SCX biphasic column and a RP separation column (Li et al., 2009). The SCX segment of the biphasic column was a monolithic column (200 μM i.d.) as a plug and the RP segment was a capillary column packed with C_18_ AQ beads (5 μM, 120 Å). Also, the separation column was a 75 μM i.d. capillary column in-house packed with C_18_ AQ beads (3 μM, 120 Å). Flow rate of the LC–MS/MS system was adjusted to about 200 nl/min. The peptides or phosphopeptides were firstly loaded onto the RP segment of the biphasic column in order to reduce the sample loss, and then were transferred into the SCX segment under a RP gradient (0 mM NH_4_Ac). A series of stepwise increasing salt concentrations were used to elute the peptides to second dimensional RP separation column. Mobile phase 0.1% FA aqueous solution (buffer A) and 0.1% FA acetonitrile solution (buffer B) were used for RPLC separation. 1,000 mM NH_4_Ac (buffer C, pH 2.7) and 100 mM NH_4_Ac (buffer D, pH 2.7) were applied to form the salt concentration.

For proteomics analysis, the salt concentrations were 0 mM, 50 mM, 100 mM, 200 mM, 300 mM, 400 mM, 500 mM, and 1,000 mM NH_4_AC. The RP gradient elution was performed as follows: 2–5% B for 2 min; 5–35% B for 150 min; 35–90% B for 3 min; 90% B for 10 min and finally equilibration with mobile phase A for 20 min. For phosphoproteomics analysis, because of the hydrophilic ability of the phosphopeptides, the salt concentrations were 0 mM, 10 mM, 20 mM, 30 mM, 50 mM, 75 mM, 100 mM, 200 mM, and 1, 000 mM NH_4_Ac. The RP gradient was performed with a gradient of 0–2% B in 2 min; 2–25% B in 90 min; 25–35% B in 5 min; 35–80% B in 3 min; 80% B in 10 min and 100% A in 20 min. Each salt fraction lasted 10 min followed by a 15 min equilibrium with buffer A. For biological replicates, all the procedures were the same as describe above.

The mass spectrometer was operated in data-dependent MS/MS acquisition mode. The parameters were set as: ion spray voltage, 2.2 kV; ion transfer capillary, 250°C; full mass scan, 400–2000 m/z; resolution, 60,000 at m/z 400; the 20 most intense precursors were selected to fragmentation *via* collision induced dissociation (CID) at a minimum signal intensity 300; and the multistage activation was enabled for phosphopeptides analysis. The dynamic exclusion function was set as follows: repeat count 1; duration 30 s; exclusion list size 500; exclusion duration 90 s.

### Quantification by MaxQuant

All the RAW files were searched by software MaxQuant version 1.3^1^ against a NCBI yeast protein database, which has 10,957 sequences and was downloaded from website.^2^ For phosphopeptides database searching, oxidation on methionine (+15.9949 Da), phosphorylation on serine, threonine and tyrosine (+79.96633 Da) were set as variable modification, while for total protein analysis, only oxidation (M) was set as variable modification. Carbamidomethylation on cysteine (+57.0215 Da) was set as fixed modification for both analyses. Triplets were selected as quantification mode and the dimethyl Lys 0, 4, 8, and dimethyl N-termini 0, 4, 8 were set as light, intermediate and heavy labels, respectively. Mass tolerance for precursor ion was 10 ppm and 0.5 Da for fragment ion. Specific trypsin (KR/P) was set as the enzyme and up to two missed cleavage sites were allowed. The false discovery rates (FDRs) for both peptide and protein identifications were set to 0.01. All the other parameters in MaxQuant used Default settings.

### Mapping reads to the reference genome

As the raw reads which were transferred from base calling may contain low quality reads and/or adaptor sequences, preprocessing is necessary before starting further analysis. Clean reads were mapped to *R*. *toruloides* NP11 reference gene sequences (Zhu et al., 2012) and to *R*. *toruloides* reference genome sequences set using SOAP aligner/SOAP2 (Li et al., 2009). No more than two mismatches were allowed in the alignment.

### Atg9-RNAi: Strains, plasmids and media

Strains and plasmids used in this study are summarized in Table 1. The RNAi strains such as Atg9-RNAi-1, 2 and 3 were obtained from the NP11 by delivered the RNAi vector (Supplementary Figure S1). *Escherichia coli* DH5α was routinely used for gene cloning and manipulation. The plasmid pZPK-P_PGK_-HYG-T_NOS_-P_GPD_-Atg9-1-sen-Atg9-1-anti-T_HSP_ and pZPK-P_PGK_-HYG-T_NOS_-P_GPD_-Atg9-2-sen-Atg9-2-anti-T_HSP_ were constructed and stored in our laboratory, with the first and second exon of Atg9 from the genome DNA of NP11.

**Table 1:** Strains and plasmids used in this study.

Strain or plasmid	Relevant characteristics	Source
Strains		
*Rhodotorula toruloides* NP11	*MAT A*, haploid strain	This lab
*Rhodotorula toruloides* CGMCC 2.1389	Diploid strain	CGMCC
*Agrobacterium tumefaciens* AGL1	*AGL0 recA::bla pTiBo542△T Mop*^+^ *CbR*	Lazo et al. (1991)
AGL1-HYG-pGPD -MCS	AGL1/pZPK-P_PGK_-HYG-T_NOS_-P_GPD_-MCS-T_HSP_	This lab
*Escherichia coli* DH5α	F^−^, φ80d*lacZ*ΔM15, Δ(*lacZYA*-*argF*)U169, *deoR*, *recA1*, *endA1*, *hsdR17*(*rK*^−^, *mK^+^*), *phoA*, *supE44*, *λ^−^*, *thi-1*, *gyrA96*, *relA1*	TaKaRa
*Rhodotorula toruloides* Atg9-RNAi-1	NP11/ pZPK-P_PGK_-HYG-T_NOS_-P_GPD_-Atg9-1-sen-Atg9-1-anti -T_HSP_	This study
*Rhodotorula toruloides* Atg9-RNAi-2	NP11/ pZPK-P_PGK_-HYG-T_NOS_-P_GPD_-Atg9-2-sen-Atg9-2-anti -T_HSP_	This study
*Rhodotorula toruloides* Atg9-RNAi-3	NP11/ pZPK-P_PGK_-HYG-T_NOS_-P_GPD_-Atg9-1-sen-Atg9-1-anti -T_HSP_	This study
Plasmids		
pZPK-P_PGK_-HYG-T_NOS_-P_GPD_-MCS-T_HSP_	Kan^R^	This study
pZPK-P_PGK_-HYG-T_NOS_-P_GPD_-Atg9-1-sen-Atg9-1-anti-T_HSP_	Kan^R^	This study
pZPK-P_PGK_-HYG-T_NOS_-P_GPD_-Atg9-2-sen-Atg9-2-anti-T_HSP_	Kan^R^	This study

To construct the RNAi vector, two reverse complement fragments of the target sequence was amplificated, respectively. To clone the forward and reverse segments of the first exon of Atg9 gene, we used the primer RtAtg9-1-sen-F, RtAtg9-1-sen-R-NcoI and RtAtg9-1-anti-F, RtAtg9-1-anti-R-NcoI summarized in Supplementary Table S1 in PCR amplification, respectively. The product of the primer pair RtAtg9-1-sen-F, RtAtg9-1-sen-R-NcoI contain the first intron as the loop of the RNAi fragment. Then we cut the end with *Nco*I (Takara, Dalian, China) and then used the DNA Ligation Kit Ver.2.1 (Takara, Dalian, China) to connect two fragments. We made the connected fragment and the plasmid pZPK-P_PGK_-HYG-T_NOS_-P_GPD_-MCS-T_HSP_ enzyme-cut with *Eco*RV and *Spe*I and link up as a new vector named pZPK-P_PGK_-HYG-T_NOS_-P_GPD_-Atg9-1-sen-Atg9-1-anti-T_HSP._ As the process we construct the vector pZPK-P_PGK_-HYG-T_NOS_-P_GPD_-Atg9-2-sen-Atg9-2-anti-T_HSP_ with the primer RtAtg9-2-sen-F, RtAtg9-2-sen-R-*Nco*I and RtAtg9-2-anti-F, RtAtg9-2-anti-R-*Nco*I and the RNAi target is the second exon of Atg9 gene. The constructed vector transformed into *E*. *coli* DH5α for gene cloning.

The two RNAi vector were digested by *Spe*I and *Eco*RV together for identification. The DNA fragments were run on 1.0% agarose gel with GoldView (Dingguo, Beijing, China) in 0.5 × TBE buffer. The recombinant plasmid was cut out of a fragment of 1.6 kb with *Spe*I and *Eco*RV (Supplementary Figure S1), which equaled double sizes of the first exon of Atg9 gene plus one intron as the loop. According to the electrophoresis map, we got three correct plasmids. We transformed the vector into the *Agrobacterium tumefaciens* AGL1 by electrotransformation. Finally, we got three RNAi engineered strains by *Agrobacterium*-mediated transformation (ATMT; Lin et al., 2014). Yeast strains were grown in PL and PR medium supplemented with trace element solution as described at the second part of chemostat cultivations: strains and media.

### RT-PCR

As the RNA extraction process, we got the RNA of the RNAi strains and wild type (WT) NP11. RT-PCR was employed to validate the decrease or disappear of Atg9 mRNA with PrimeScript™ II High Fidelity RT-PCR Kit (Takara, Dalian, China). The products of RT-PCR was run on 1.0% agarose gel in 0.5 × TBE buffer. The test primers in RT-PCR were the same in the process of vector construction.

### Live-cell fluorescence microscopy

Yeast cells cultured in PL and PR culture medium were stained with LysoTracker dye (Invitrogen, CA, United States). The loading pieces of RNAi and WT strains after staining were observed through fluorescent microscope. Images were captured using NIS Elements.

### Total sugar content and lipid content

Glucose was measured using an SBA-40D glucose analyzer (Shandong Academy of Sciences, Jinan, China). Cells from 30 mL cultures broth were harvested by centrifugation at 8,000 g for 5 min and washed twice with distilled water.

To obtain dry cell weight (DCW) and lipid content, methods reported before were implemented (Li et al., 2007). Lipid content was expressed as gram lipid per gram DCW. Lipid yield was calculated as gram lipid produced per gram sugar consumed.

## Results

### Chemostat cultures

To study the influence of Pi limitation stress on *R*. *toruloides*, we grew chemostat cultures of *R*. *toruloides* using minimal medium with 27 g l^−1^ glucose but different Pi loadings to ensure initial carbon-to-phosphorus (C/P) molar ratio of 63 and 2,164, and samples were coded as F3 and P0, respectively. After chemostat cultures, cells accumulated lipids to 7.3% and the residual Pi concentration was 7.35 mM in F3. In P0, the cellular lipid content was 43.9% and the residual Pi concentration was under the detection limit. These results were reported by our lab and clearly indicated that Pi limitation significantly promoted lipid accumulation (Wang et al., 2018).

### Quantitative proteome and phosphoproteome analysis

To describe the molecular changes underlying the observed phenotype, an in-depth quantitative profile of *R*. *toruloides* phosphoproteomics was performed. As it is important to increase the number of quantified peptides for improved protein quantification coverage and accuracy, we fractionated each sample into 8 (proteomics) or 9 fractions (phosphoproteomics) by on-line 2D SCX-RP system for MS analysis, which reduced the complexity of each fraction while maintained a global peptide screen. While for phosphoproteomics profiling, multiple technical or biological replicate analyses were often adopted to control the quantification accuracy, as a phosphorylation site was quantified by a single phosphopeptide. Therefore, biological triplicates were conducted for proteomics and phosphoproteomics analysis.

It is crucial for quantification analysis to guarantee a high-quality profile. Only the proteins and phosphorylation sites quantified in at least two biological replicates were counted. The results were further filtered by the coefficient of variation (CV) of the biological replicates less than 50%. As for phosphoproteomics analysis, the high quality of phosphorylation sites were also satisfied with the criteria of phosphorylation site localization probability >0.75 (class I phosphorylation sites) and phosphorylation site score difference ≥ 5. A total of 3,556 proteins were identified and 460 proteins were found up-regulated in P0 sample and 782 were found down-regulated (relative expression ≥1.2 or ≤ 0.8) when a threshold of 1.2-fold up or down were considered to show a significant expression change. In addition, a total of 4,280 phosphopeptides and 5,659 phosphosites belonging to 1,649 phosphoproteins, were identified through analysing the phosphoproteome (Supplementary Tables S2–S4). A threshold of 1.2-fold up or down in phosphoproteomics analysis were selected, 890 and 708 phosphosites were found up-regulated and down-regulated in P0 sample, respectively (apart from the changes of proteins themselves.

### Phosphorylation motif discovery

**Figure 2:**
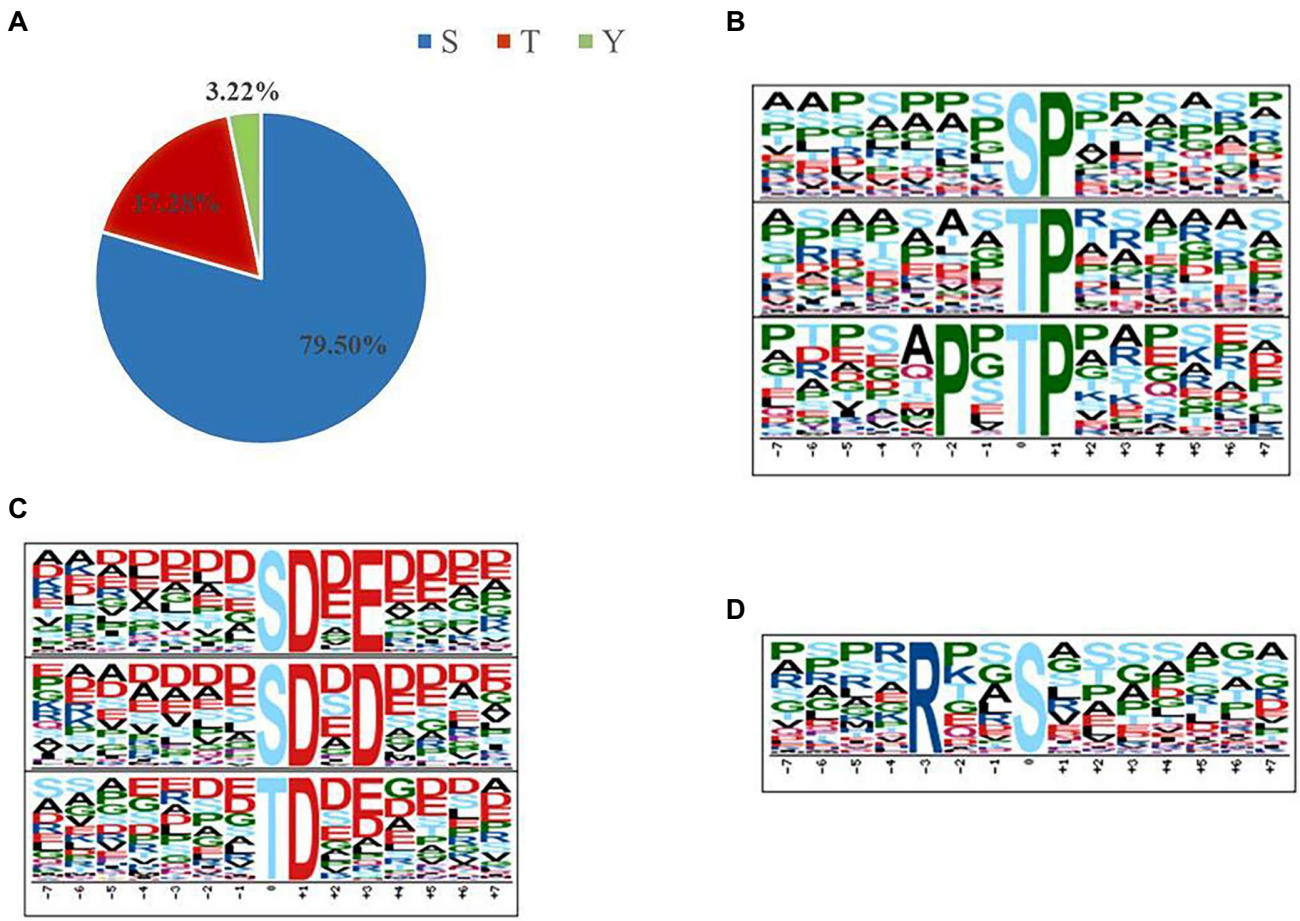
Phosphorylation-specific motifs using the Motif-X algorithm (Schwartz and Gygi, 2005). The complete set of motifs is shown in Supplementary Table S5. **(A)** In total, 5,659 phosphosites (4,499 pSer, 978 pThr, and 182 pTyr) were identified. **(B,C)** Sequence logos for some examples of single-phosphorylation motifs where the phosphorylated residue (S or T) is centered. **(B)** Pro-directed motifs. **(C)** Acidic motifs. **(D)** Basic motif.

The distribution of ±7 amino acids (AA) surrounding the 5,659 phosphosites were shown, and the sequence logo was created by WebLogo online software^3^ for Ser, Thr, and Tyr, respectively (Supplementary Figure S2). Given that protein phosphorylation appears to be an important feature of the proteotype, motifs associated with localized phosphorylation sites were identified using Motif-X^4^ (Schwartz and Gygi, 2005). To do this, 4,499 pSer, 978 pThr, and 182 pTyr were all aligned, and their lengths were also adjusted to 7 AA from the central position. Supplementary Table S5 lists the motifs generated containing a minimum of 50 pSer, 20 pThr and 3 pTyr occurrences. In total, 29 phosphoserine motifs, 18 phosphothreonine motifs, and none phosphotyrosine motif were identified. The phosphoserine motifs could be grouped into three major classes: Pro-directed, basic, and acidic. Meantime, we did not discovery basic motif in phosphothreonine motifs (Figure 2). Interestingly, the phosphoserine motifs were consistent between the samples under Pi limitation and Pi repletion.

Certain motifs are commonly associated with specific kinases (Peri et al., 2004) and were prevalent in our data set (Supplementary Table S5). Proline-directed motifs (sP and tP) were recognized by cyclin-dependent kinase 5 (Cdk5; Pinna and Ruzzene, 1996). Besides, PxtP recognized by MAP kinase was well represented in our data, with substrates including MAPK2 and several Rsk family members (Ballif et al., 2005). Basophilic kinase motifs such as Rxxs (PKA) were identified (Huang et al., 2007). In addition, several acidic casein kinase II (CK2) motifs (e.g., sDxE, sDxD, and tD) were identified in our data set.

### Intracellular regulators associated with autophagy analysis

Intracellular Pi levels are maintained by the Pi responsive signaling (*PHO*) pathway that modulates expressions of Pi-responsive genes. In the metabolism of phosphorus, Pho85 (RHTO_01208), is best known for its pivotal role in the *PHO* pathway, a signaling pathway that coordinates the responses of yeast to Pi-starvation (Carroll and O’Shea, 2002). Autophagy is an important aspect of cells survival strategy against stressful conditions, and it would be activated under Pi-limitation.

**Figure 3:**
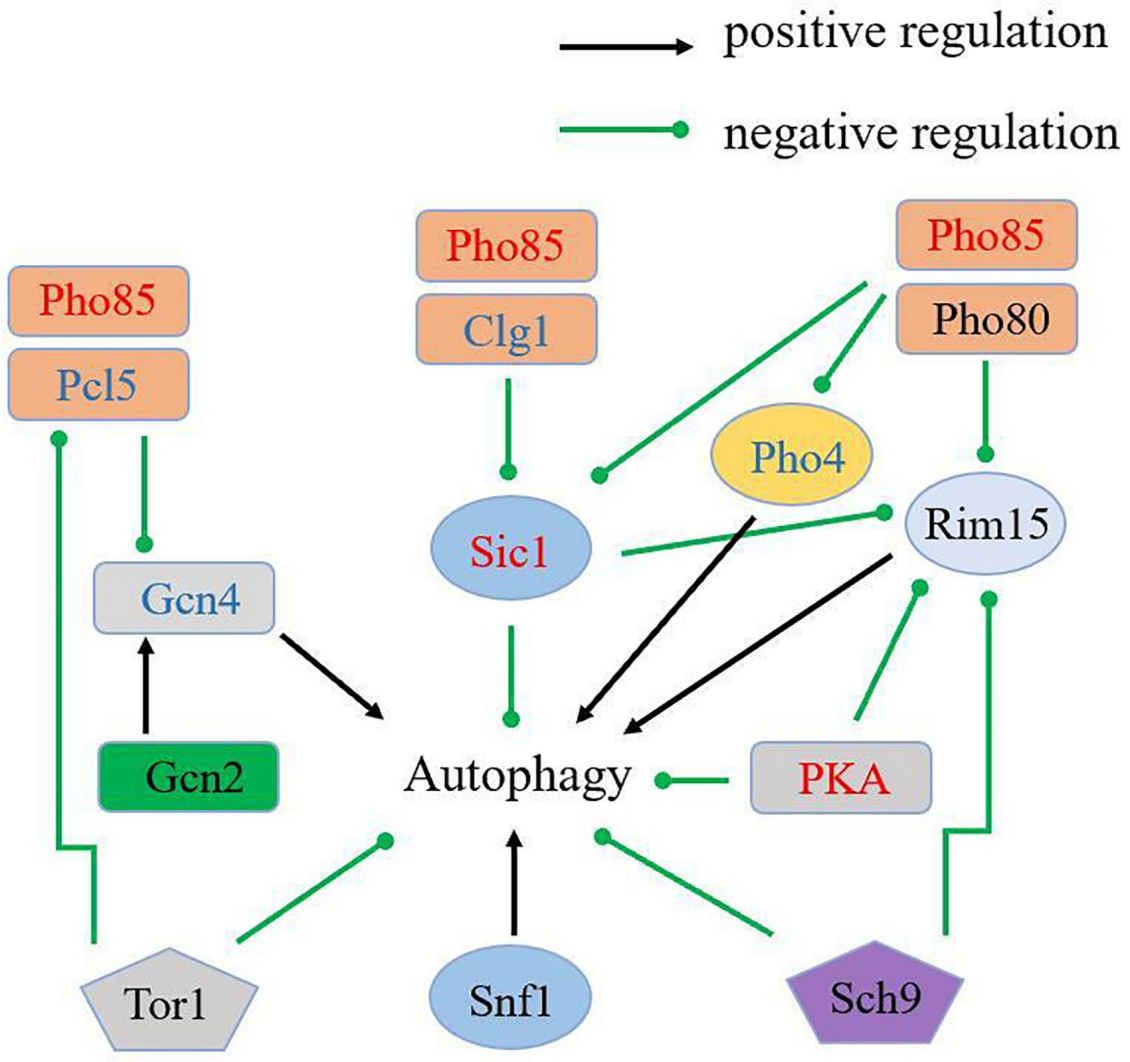
Schematic overview of the key components in autophagy regulation and the regulation of proteins in proteome. Up-regulated proteins were in red; not changed proteins were in black; not detected proteins were in blue.

Pho85 negatively regulates starvation-induced autophagy antagonistically with a positive regulator of autophagy, Snf1, the closest yeast homolog of the mammalian AMP-activated protein kinase (AMPK; Wang et al., 2001). The cyclins Pho80 and Pcl5, in concert with Pho85, negatively regulate autophagy through downregulating the protein kinase Rim15 and the transcription factors Pho4 and Gcn4. When complexed with the cyclins Clg1 and Pho80, Pho85 positively regulates autophagy through promoting the degradation of Sic1, a negative regulator of autophagy that targets Rim15 (Yang et al., 2010). In addition, the rapamycin-sensitive TORC1, the Ras/cAMP-dependent protein kinase A (PKA) signaling pathway, and Sch9, a homolog of mammalian protein kinase B (PKB)/Akt also negatively regulate autophagy (Budovskaya et al., 2004; Schmelzle et al., 2004; Yorimitsu et al., 2007; Chang and Neufeld, 2009). In contrast, the Gcn2 kinase pathway is involved in the positive regulation of autophagy (Talloczy et al., 2002). To explore autophagy regulation under Pi-limitation, we focused on PHO proteins and many protein kinases which play roles in the regulation of autophagy, including TORC1, PKA and Sch9 (Figure 3). Interestingly, autophagy inhibitory proteins Sic1 (RHTO_02516), PKA (RHTO_02603) and Pho85 were all up-regulated expression in proteomics, which repressed autophagy under Pi-limitation (Supplementary Table S2). Moreover, Pho80 (RHTO_03958), Rim15 (RHTO_04687), Gcn2 (RHTO_01618), Sch9 (RHTO_07033), Snf1 (RHTO_04494), TORC1 (RHTO_05832) were not changed. Pho4 (RHTO_00753), Gcn4 (RHTO_01666), Pcl5 (RHTO_02398) and Clg1 (RHTO_08156) were not detected (Supplementary Table S3). These data suggest that autophagy may not be induced by proteins associated with the metabolism of phosphorus. We therefore concluded that PSiA was not under the control of the *PHO* pathway.

In proteomics, 109 of the 112 genes being annotated as ribosome structural components were down-regulated expression (Supplementary Table S2). This suggests that ribosome synthesis is suppressed and superfluous ribosome is degraded under Pi-limitation, providing more Pi for cell survival.

### Autophagy-related proteins analysis

As a major intracellular degradation pathway, autophagy is tightly regulated to prevent cellular dysfunction in all eukaryotic cells. In proteomics and protein phosphorylation quantitative analysis, autophagy-related (Atg) proteins were analyzed (Table 2). We found that Atg5 (RHTO_06785), Atg7 (RHTO_00622), Atg8 (RHTO_06526), Atg11 (RHTO_00852), Atg24 (RHTO_04976) and Atg27 (RHTO_06153) were upregulated in proteomics under Pi-limitation (Supplementary Table S2). Interestingly, the Atg13 (RHTO_02383) and Atg9 (RHTO_06411) were significantly phosphorylated in phosphoproteome, while they had not been detected in proteome under Pi-limitation (Table 2; Supplementary Table S4). In previous reports, Atg13 was dephosphorylated rapidly in response to nitrogen starvation and slowly under Pi-starvation in *S*. *cerevisiae* (Suzuki et al., 2015; Yokota et al., 2017). As the pivotal regulator of autophagy induction, Atg1-dependent Atg9 phosphorylation regulated autophagy (Papinski and Kraft, 2014). In view of the above-mentioned facts, as the only transmembrane Atg protein, Atg9 has been regarded as key to one of the major driving forces of autophagy (Munakata and Klionsky, 2010). We suppose that Atg9 play a crucial role in response to Pi starvation, and the influence of Atg9 on autophagy and lipid accumulation in *R*. *toruloides* was further investigated.

**Table 2:** List of the regulation of autophagy-related proteins in proteome and phosphoproteomics.

Name	Accession	Annotation	Change in proteome (P0:F3)	Change in phosphorproteomics (site; P0:F3)
Atg3	RHTO_01636	Autophagy-related protein 3	0.67	NaN
Pcl5	RHTO_06785	Autophagy-related protein 5	1.72	NaN
Atg7	RHTO_00622	Autophagy-related protein 7	1.38	NaN
Atg8	RHTO_06526	GABA(A) receptor-associated protein	2.00	NaN
Atg9	RHTO_06411	Autophagy-related protein 9	NaN	4.85 (307); 2.57 (128)
Atg11	RHTO_00852	Autophagy-related protein 11	1.36	NaN
Atg12	RHTO_01037	Autophagy-related protein 12	1.11	NaN
Atg13	RHTO_02383	Autophagy-related protein 13	NaN	2.27 (553)
Atg18	RHTO_02334	WD repeat domain phosphoinositide-interacting protein	1.28	NaN
Atg20	RHTO_04181	Sorting nexin-41	0.67	NaN
Atg24	RHTO_04976	Lipid binding protein	1.72	NaN
Atg26	RHTO_07138	Sterol 3-beta-glucosyltransferase, glycosyltransferase family 1 protein	1.38	NaN
Atg27	RHTO_06153	Autophagy-related protein 27	2.00	NaN

NaN, not detectable.

### Autophagy and lipid accumulation influenced by Atg9

**Figure 4:**
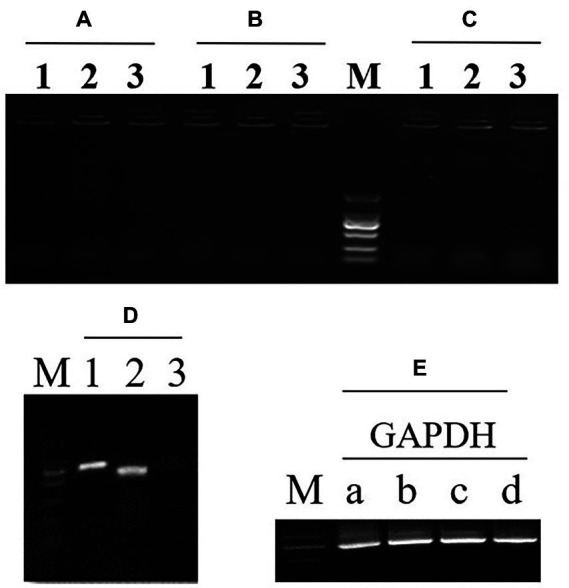
RT-PCR detection of the first (1) and second (2) exon in *ATG9* gene and the first exon with intron (3) to detect the gDNA. **(A–C)** Three *ATG*9 RNAi strains. **(D)** The wild type strain NP11. **(E)** There is an endogenous gene GAPDH as a reference.

To investigate whether Atg9 is required in autophagy pathways, Atg9-RNAi strains was constructed: firstly, the recombinant plasmids was verified by digestion by *Spe*I and *Eco*RV together (Supplementary Figure S3). Secondly, the vector was transferred to *R*. *toruloides* NP11 by ATMT. Three *ATG*9 RNAi engineered strains were selected randomly and *R*. *toruloides* NP11 was selected as the WT. At the end, RT-PCR was implemented and the electrophoresis map is shown in Figure 4.

**Figure 5:**
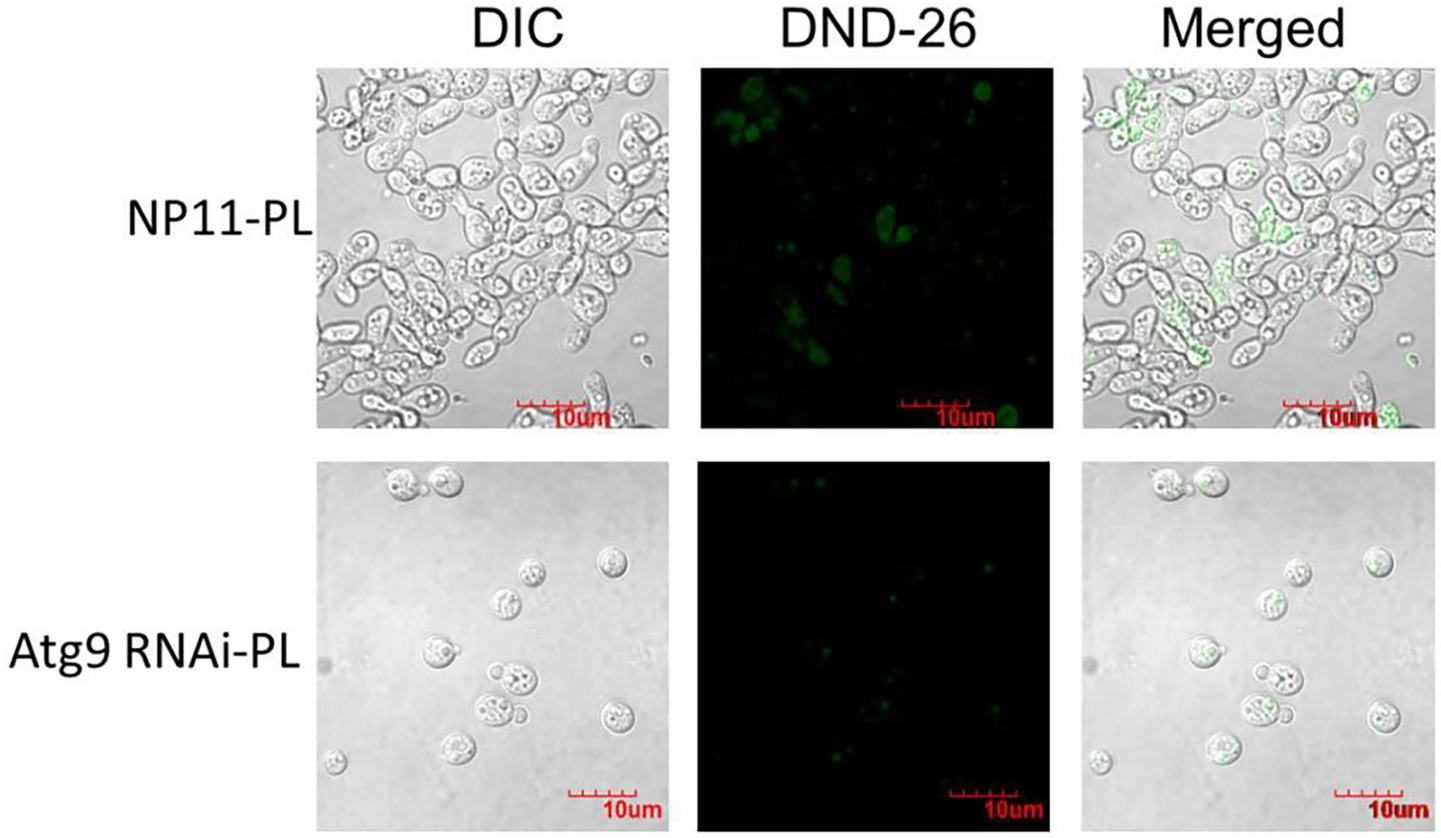
The *R*. *toruloides* WT and RNAi strains cultured in the PL medium with the fluorescence microscope after LysoTracker® Green DND-26 staining.

**Figure 6:**
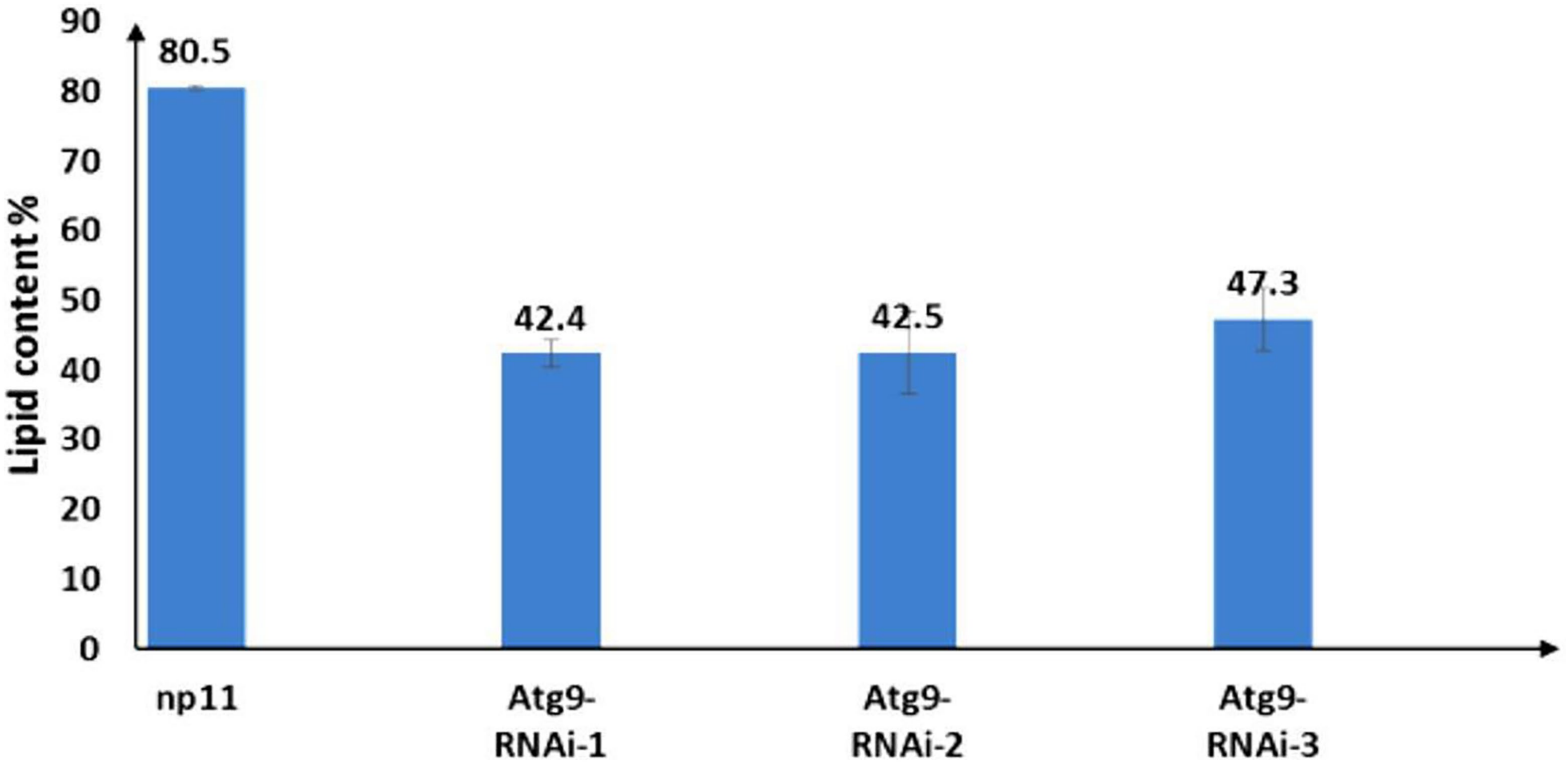
Lipid content of *R*. *toruloides* WT and *ATG*9 RNAi silencing cells under phosphorus limitation conditions.

The autophagosome of RNAi strains and the WT NP11 were recorded by fluorescent microscope and the images were captured using NIS Elements. As shown in Figure 5, under starvation conditions, autophagy was induced in NP11 cells but not in *ATG*9-silencing cells. These data suggested that Atg9 plays important roles in the formation of autophagosome under Pi limitation. At the same time, total lipids of NP11 cells and *ATG*9-silencing cells were extracted by the established method (Li et al., 2007), and lipid contents were estimated. It was found that the lipid content of *ATG*9-silencing cells was significantly lower than that of NP11 cells cultured under Pi limitation conditions (Figure 6). These results suggested that lipid content was reduced as autophagy was suppressed and there was a very close relationship between lipid accumulation and autophagy.

## Discussion

In order to study the influence of Pi limitation on autophagy in oleaginous yeast and the relationship between autophagy and lipid accumulation, here we implemented proteomic and phosphorylation quantitative analysis of *R*. *toruloides* under Pi limitation and Pi replete conditions.

Phosphate limitation culture has been practiced in various species for further research (Wu et al., 2010; Kamlesh et al., 2016; Wang et al., 2018; Salmanzadeh et al., 2020). Moreover, Pi limitation leads to phosphoric acid starvation in the cytosol and autophagy would be activated against stressful conditions to maintain cell survival. In previous report, upon nitrogen starvation, ribosome synthesis is immediately stopped and superfluous ribosomes are degraded (Zhu et al., 2012). The expression of ribosomal proteins was significantly down-regulated in P0 sample. This, in turn, led to the repression of ribosome biogenesis and degradation of superfluous ribosomes. During ribosome degradation, not only ribosomal proteins, but also a large amount of ribosomal RNAs should be degraded, providing more Pi for cell survival. Under nutrient starvation conditions, TORC1 is inhibited and autophagy is induced in *S*. *cerevisiae* (Chang and Neufeld, 2009). PSiA was regulated by TORC 1, but not by the PHO pathway in *S*. *cerevisiae* (Yokota et al., 2017). At a low Pi concentration, Pho81 inhibits Pho85 to prevent the transcriptional activator Pho4 from phosphorylation, resulting in its nuclear localization and transcriptional activation of PHO genes (Oshima et al., 1996). Although Pho85 does not play a major role in regulating autophagy compared to the TORC1 complex, the multifunctional Pho85 is critical to ensure appropriate autophagy activity during intracellular stress conditions (Yang et al., 2010). We first investigated whether PHO proteins and protein kinases associated with the regulation of autophagy could be involved in PSiA in *R*. *toruloides*.

We found that the protein levels of Pho85, Sic1, and PKA were all up-regulated, which repressed autophagy. In addition, Pho80, Rim15, Gcn2, Sch9, Snf1, TORC1 were not changed. Pho4, Gcn4, Pcl5 and Clg1 were not detected. We therefore concluded that PSiA was not under the control of the *PHO* pathway, which was consistent with PSiA in *S*. *cerevisiae* (Yokota et al., 2017). However, PSiA in *S*. *cerevisiae* was found regulated by Tor1, while, in *R*. *toruloides*, the level of Tor1 was not changed under PSiA.

**Figure 7:**
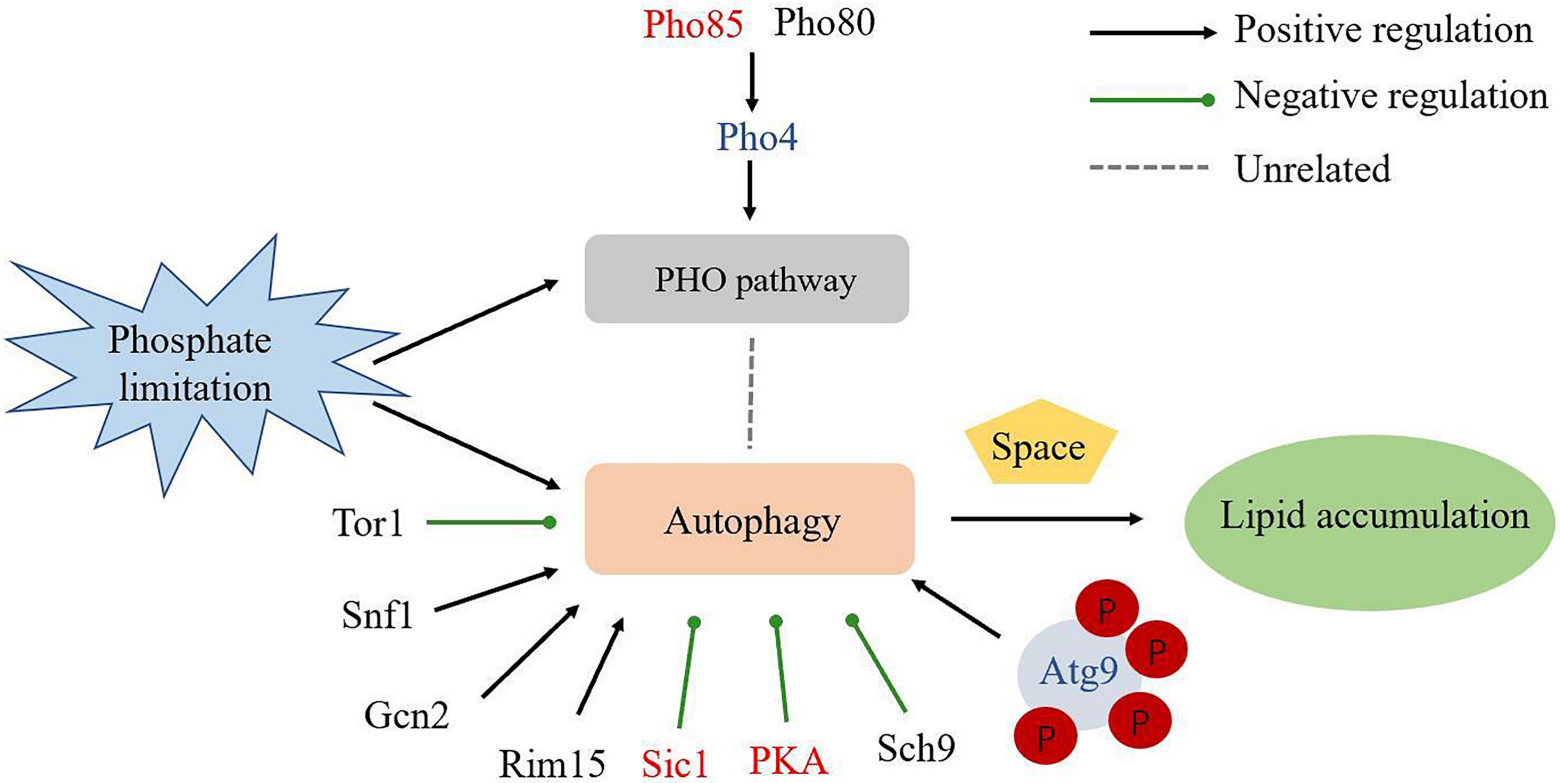
Schematic overview of the regulation of autophagy and lipid accumulation under phosphate limitation in *R*. *toruloides*. Up-regulated proteins were in red; not changed proteins were in black; not detected proteins were in blue.

Then, we focused on Atg proteins in proteome and phosphoproteome under Pi-limitation. Atg5, Atg7, Atg8, Atg11, Atg24 and Atg27 were upregulated in proteome, Atg13 and Atg9 were significantly phosphorylated in phosphoproteome. Therefore, we concluded that PSiA was regulated by Atg proteins under Pi-limitation in *R*. *toruloides* (Figure 7). As the only transmembrane Atg protein, Atg9 has been regarded as key to one of the major driving forces of autophagy (Munakata and Klionsky, 2010). We speculated that Atg9 played a major role in the form of autophagosome in *R*. *toruloides*. *ATG*9 RNAi silencing strains was constructed and cultivated under Pi-limitation condition. Comparing to the WT strain, autophagic response was repressed in RNAi strains of *R*. *toruloides* and the lipid content was also reduced. Our results showed that Atg9 was critical to ensure appropriate autophagy activity and lipid accumulation during Pi limitation in *R*. *toruloides*. When *ATG*9 was knocked down, the engineered *R*. *toruloides* strain produced significantly less lipids under Pi-limitation, suggesting that autophagy required Atg9 in *R*. *toruloides* and autophagy was conducive to lipid accumulation. We speculate that autophagy removes dysfunctional cellular components to provide more space for lipid accumulation in oleaginous yeast.

Moreover, recent researches show that knock out or knock down of ATG9A in human cells results in increased number and size of lipid droplets and knock out of the orthologous Atg-9 in *C*. *elegans* also causes an increase in the size of lipid droplets in hypodermal cells (Mailler et al., 2021). This observation may be due to the differences between the species and the physiological status-i.e. the *R*. *toruloides* was under Pi- limitation, but not the *C*. *elegans*. Past researches showed that *ATG*9 RNAi silencing could reduce autophagic response (Nagy et al., 2014) and knock out of Atg9 appeared to cause dysregulation of lipid metabolism and oxidative phosphorylation (Xiong et al., 2021).

Phosphoproteins and certain motifs were identified on the phosphoproteins, certain motifs associated with specific kinases (Peri et al., 2004) were prevalent in our data set and none of new phosphorylation motifs were detected. Between Pi-replete and Pi-limited conditions, phosphorylation motifs were the same, showing protein phosphorylation were regulated rigorously even Pi was deficient.

In conclusion, we documented the proteome and phosphoproteome of the oleaginous yeast *R*. *toruloides* under Pi-limitation conditions. It was found that ribosome structural genes were down-regulated and the Atg-proteins Atg9 was upregulated significantly leading to activate autophagy. In addition, Atg11 was upregulated, suggesting some roles in bulk autophagy. Our experiment showed that RNAi silencing of ATG9 could reduce autophagic response and lipid production in *R*. *toruloides*. Our data supported that PSiA could be regulated by Atg proteins, but not the *PHO* pathway and Tor1. Intuitively, autophagy may remove dysfunctional cellular components to provide more space and resources for lipid accumulation in oleaginous yeast (Figure 7). The information provides new foundation to guide further mechanistic study of yeast oleaginicity and genetic engineering for advanced lipid producing cell factory.

## Data availability statement

The datasets presented in this study can be found in online repositories. The names of the repository/repositories and accession number(s) can be found in the article/Supplementary material.

## Author contributions

Z-bZ, M-lY, and S-fZ conceived the study, designed the experiments, and revised the manuscript. Y-nW and F-jL analyzed proteome and phosphoproteome data, showed the results, and wrote the manuscript. Y-nW, H-dL, YZ, and XJ conducted the experiments. All authors contributed to the article and approved the submitted version.

## Funding

This work was financially supported by National Natural Science Foundation of China (Grant Nos. 31870042, and 21721004).

## Conflict of interest

The authors declare that the research was conducted in the absence of any commercial or financial relationships that could be construed as a potential conflict of interest.

## Publisher’s note

All claims expressed in this article are solely those of the authors and do not necessarily represent those of their affiliated organizations, or those of the publisher, the editors and the reviewers. Any product that may be evaluated in this article, or claim that may be made by its manufacturer, is not guaranteed or endorsed by the publisher.

## Footnotes

^1^ http://www.maxquant.org/

^2^ http://www.ncbi.nlm.nih.gov/

^3^ http://weblogo.berkeley.edu/logo.cgi

^4^ http://motif-x.med.harvard.edu

## Abbreviations

AA: amino acids
AKT: serine/threonine kinase
ATG: autophagy related
ATMT: Agrobacterium-mediated transformation
CDK: cyclin-dependent kinase
CID: collision induced dissociation
DCW: dry cell weight
DTT: dithiothreitol
FDRs: false discovery rates
IMAC: immobilized metal ion affinity chromatography
PHO: phosphate responsive signaling
PSiA: Pi starvation-induced autophagy
RP-SCX-RP: phase-strong cation exchange-reversed phase
RT-PCR: Reverse transcription-polymerase chain reaction
SCX: strong cation exchange
TORC: target of rapamycin complex
WT: wild type

## References

[ref1] Ballif B. A. Roux P. P. Gerber S. A. Mac Keigan J. P. Blenis J. Gygi S. P. (2005). Quantitative phosphorylation profiling of the ERK/p 90 ribosomal S6 kinase-signaling cassette and its targets, the tuberous sclerosis tumor suppressors. Proc. Natl. Acad. Sci. U. S. A. 102, 667–672. doi: 10.1073/pnas.0409143102, PMID: 15647351 PMC545566

[ref2] Beausoleil S. A. Jedrychowski M. Schwartz D. Elias J. E. Villen J. Li J.. (2004). Large-scale characterization of HeLa cell nuclear phosphoproteins. Proc. Natl. Acad. Sci. U. S. A. 101, 12130–12135. doi: 10.1073/pnas.0404720101, PMID: 15302935 PMC514446

[ref3] Budovskaya Y. V. Stephan J. S. Reggiori F. Klionsky D. J. Herman P. K. (2004). The Ras/cAMP-dependent protein kinase signaling pathway regulates an early step of the autophagy process in *Saccharomyces cerevisiae*. J. Biol. Chem. 279, 20663–20671. doi: 10.1074/jbc.M400272200, PMID: 15016820 PMC1705971

[ref4] Carroll A. S. O’Shea E. K. (2002). Pho85 and signaling environmental conditions. Trends Biochem. Sci. 27, 87–93. doi: 10.1016/s0968-0004(01)02040-0, PMID: 11852246

[ref5] Chang Y. Y. Neufeld T. P. (2009). An Atg1/Atg13 complex with multiple roles in TOR-mediated autophagy regulation. Mol. Biol. Cell 20, 2004–2014. doi: 10.1091/mbc.E08-12-1250, PMID: 19225150 PMC2663935

[ref6] Cherra S. J. 3rd Kulich S. M. Uechi G. Balasubramani M. Mountzouris J. Day B. W.. (2010). Regulation of the autophagy protein LC3 by phosphorylation. J. Cell Biol. 190, 533–539. doi: 10.1083/jcb.201002108, PMID: 20713600 PMC2928022

[ref7] Ficarro S. B. McCleland M. L. Stukenberg P. T. Burke D. J. Ross M. M. Shabanowitz J.. (2002). Phosphoproteome analysis by mass spectrometry and its application to *Saccharomyces cerevisiae*. Nat. Biotechnol. 20, 301–305. doi: 10.1038/nbt0302-301, PMID: 11875433

[ref8] Gruhler A. Olsen J. V. Mohammed S. Mortensen P. Faergeman N. J. Mann M.. (2005). Quantitative phosphoproteomics applied to the yeast pheromone signaling pathway. Mol. Cell. Proteomics 4, 310–327. doi: 10.1074/mcp.M400219-MCP200, PMID: 15665377

[ref9] Hu C. Zhao X. Zhao J. Wu S. Zhao Z. K. (2009). Effects of biomass hydrolysis by-products on oleaginous yeast *Rhodosporidium toruloides*. Bioresour. Technol. 100, 4843–4847. doi: 10.1016/j.biortech.2009.04.041, PMID: 19497736

[ref10] Huang D. Friesen H. Andrews B. (2007). Pho85, a multifunctional cyclin-dependent protein kinase in budding yeast. Mol. Microbiol. 66, 303–314. doi: 10.1111/j.1365-2958.2007.05914.x, PMID: 17850263

[ref11] Huang Q. Wang Q. Gong Z. Jin G. Shen H. Xiao S.. (2013). Effects of selected ionic liquids on lipid production by the oleaginous yeast *Rhodosporidium toruloides*. Bioresour. Technol. 130, 339–344. doi: 10.1016/j.biortech.2012.12.022, PMID: 23313678

[ref12] Kamada Y. Funakoshi T. Shintani T. Nagano K. Ohsumi M. Ohsumi Y. (2000). Tor-mediated induction of autophagy via an Apg1 protein kinase complex. J. Cell Biol. 150, 1507–1513. doi: 10.1083/jcb.150.6.1507, PMID: 10995454 PMC2150712

[ref13] Kamlesh K. Y. Neelima S. Ram R. (2016). Responses to phosphate deprivation in yeast cells. Curr. Genet. 62, 301–307. doi: 10.1007/s00294-015-0544-4, PMID: 26615590

[ref14] Kawamata T. Horie T. Matsunami M. Sasaki M. Ohsumi Y. (2017). Zinc starvation induces autophagy in yeast. J. Biol. Chem. 292, 8520–8530. doi: 10.1074/jbc.M116.762948, PMID: 28264932 PMC5437255

[ref15] Larsen M. R. Thingholm T. E. Jensen O. N. Roepstorff P. Jorgensen T. J. (2005). Highly selective enrichment of phosphorylated peptides from peptide mixtures using titanium dioxide microcolumns. Mol. Cell. Proteomics 4, 873–886. doi: 10.1074/mcp.T500007-MCP200, PMID: 15858219

[ref16] Lazo G. R. Stein P. A. Ludwig R. A. (1991). A DNA transformation-competent Arabidopsis genomic library in *agrobacterium*. Biotechnology 9, 963–967. doi: 10.1038/nbt1091-963, PMID: 1368724

[ref17] Levine B. Klionsky D. J. (2004). Development by self-digestion: molecular mechanisms and biological functions of autophagy. Dev. Cell 6, 463–477. doi: 10.1016/s1534-5807(04)00099-1 15068787

[ref18] Li R. Yu C. Li Y. Lam T. W. Yiu S. M. Kristiansen K.. (2009). SOAP2: an improved ultrafast tool for short read alignment. Bioinformatics 25, 1966–1967. doi: 10.1093/bioinformatics/btp336, PMID: 19497933

[ref19] Li Y. Zhao Z. Bai F. (2007). High-density cultivation of oleaginous yeast *Rhodosporidium toruloides* Y4 in fed-batch culture. Enzym. Microb. Technol. 41, 312–317. doi: 10.1016/j.enzmictec.2007.02.008

[ref20] Lin X. Wang Y. Zhang S. Zhu Z. Zhou Y. J. Yang F.. (2014). Functional integration of multiple genes into the genome of the oleaginous yeast *Rhodosporidium toruloides*. FEMS Yeast Res. 14, 547–555. doi: 10.1111/1567-1364.12140, PMID: 24495153

[ref21] Mailler E. Guardia C. M. Bai X. Jarnik M. Williamson C. D. Li Y.. (2021). The autophagy protein ATG9A enables lipid mobilization from lipid droplets. Nat. Commun. 12:6750. doi: 10.1038/s41467-021-26999-x, PMID: 34799570 PMC8605025

[ref22] Munakata N. Klionsky D. J. (2010). "autophagy suite": Atg9 cycling in the cytoplasm to vacuole targeting pathway. Autophagy 6, 679–685. doi: 10.4161/auto.6.6.12396, PMID: 20543572

[ref23] Nagy P. Hegedus K. Pircs K. Varga A. Juhasz G. (2014). Different effects of Atg2 and Atg18 mutations on Atg8a and Atg9 trafficking during starvation in drosophila. FEBS Lett. 588, 408–413. doi: 10.1016/j.febslet.2013.12.012, PMID: 24374083 PMC3928829

[ref24] Nakatogawa H. Suzuki K. Kamada Y. Ohsumi Y. (2009). Dynamics and diversity in autophagy mechanisms: lessons from yeast. Nat. Rev. Mol. Cell Biol. 10, 458–467. doi: 10.1038/nrm2708, PMID: 19491929

[ref25] Oda Y. Nagasu T. Chait B. T. (2001). Enrichment analysis of phosphorylated proteins as a tool for probing the phosphoproteome. Nat. Biotechnol. 19, 379–382. doi: 10.1038/86783, PMID: 11283599

[ref26] Oshima Y. Ogawa N. Harashima S. (1996). Regulation of phosphatase synthesis in *Saccharomyces cerevisiae*–a review. Gene 179, 171–177. doi: 10.1016/s0378-1119(96)00425-8 8955644

[ref27] Papinski D. Kraft C. (2014). Atg1 kinase organizes autophagosome formation by phosphorylating Atg9. Autophagy 10, 1338–1340. doi: 10.4161/auto.28971, PMID: 24905091 PMC4203558

[ref28] Papinski D. Schuschnig M. Reiter W. Wilhelm L. Barnes C. A. Maiolica A.. (2014). Early steps in autophagy depend on direct phosphorylation of Atg9 by the Atg1 kinase. Mol. Cell 53, 471–483. doi: 10.1016/j.molcel.2013.12.011, PMID: 24440502 PMC3978657

[ref29] Peri S. Navarro J. D. Kristiansen T. Z. Amanchy R. Surendranath V. Muthusamy B.. (2004). Human protein reference database as a discovery resource for proteomics. Nucleic Acids Res. 32, 497D–4501D. doi: 10.1093/nar/gkh070, PMID: 14681466 PMC308804

[ref30] Pinna L. A. Ruzzene M. (1996). How do protein kinases recognize their substrates? Biochim. Biophys. Acta 1314, 191–225. doi: 10.1016/s0167-4889(96)00083-3 8982275

[ref31] Rush J. Moritz A. Lee K. A. Guo A. Goss V. L. Spek E. J.. (2005). Immunoaffinity profiling of tyrosine phosphorylation in cancer cells. Nat. Biotechnol. 23, 94–101. doi: 10.1038/nbt1046, PMID: 15592455

[ref32] Salmanzadeh M. Sabet M. S. Moieni A. Homaee M. (2020). Heterologous expression of an acid phosphatase gene and phosphate limitation leads to substantial production of chicoric acid in Echinacea purpurea transgenic hairy roots. Planta 251:31. doi: 10.1007/s00425-019-03317-w, PMID: 31823013

[ref33] Schmelzle T. Beck T. Martin D. E. Hall M. N. (2004). Activation of the RAS/cyclic AMP pathway suppresses a TOR deficiency in yeast. Mol. Cell. Biol. 24, 338–351. doi: 10.1128/MCB.24.1.338-351.2004, PMID: 14673167 PMC303340

[ref34] Schwartz D. Gygi S. P. (2005). An iterative statistical approach to the identification of protein phosphorylation motifs from large-scale data sets. Nat. Biotechnol. 23, 1391–1398. doi: 10.1038/nbt1146, PMID: 16273072

[ref35] Suzuki S. W. Yamamoto H. Oikawa Y. Kondo-Kakuta C. Kimura Y. Hirano H.. (2015). Atg13 HORMA domain recruits Atg9 vesicles during autophagosome formation. Proc. Natl. Acad. Sci. U. S. A. 112, 3350–3355. doi: 10.1073/pnas.1421092112, PMID: 25737544 PMC4371973

[ref36] Takeshige K. Baba M. Tsuboi S. Noda T. Ohsumi Y. (1992). Autophagy in yeast demonstrated with proteinase-deficient mutants and conditions for its induction. J. Cell Biol. 119, 301–311. doi: 10.1083/jcb.119.2.301, PMID: 1400575 PMC2289660

[ref37] Talloczy Z. Jiang W. Virgin H. W. T. Leib D. A. Scheuner D. Kaufman R. J.. (2002). Regulation of starvation- and virus-induced autophagy by the eIF2alpha kinase signaling pathway. Proc. Natl. Acad. Sci. U. S. A. 99, 190–195. doi: 10.1073/pnas.012485299, PMID: 11756670 PMC117537

[ref38] Tao W. A. Wollscheid B. O'Brien R. Eng J. K. Li X. J. Bodenmiller B.. (2005). Quantitative phosphoproteome analysis using a dendrimer conjugation chemistry and tandem mass spectrometry. Nat. Methods 2, 591–598. doi: 10.1038/nmeth776, PMID: 16094384

[ref39] Wang Z. Wilson W. A. Fujino M. A. Roach P. J. (2001). Antagonistic controls of autophagy and glycogen accumulation by Snf1p, the yeast homolog of AMP-activated protein kinase, and the cyclin-dependent kinase Pho85p. Mol. Cell. Biol. 21, 5742–5752. doi: 10.1128/MCB.21.17.5742-5752.2001, PMID: 11486014 PMC87294

[ref40] Wang Y. Zhang S. Zhu Z. Shen H. Lin X. Jin X.. (2018). Systems analysis of phosphate-limitation-induced lipid accumulation by the oleaginous yeast *Rhodosporidium toruloides*. Biotechnol. Biofuels 11:148. doi: 10.1186/s13068-018-1134-8, PMID: 29849765 PMC5968551

[ref41] Wen Z. Zhang S. Odoh C. K. Jin M. Zhao Z. K. (2020). *Rhodosporidium toruloides*-a potential red yeast chassis for lipids and beyond. FEMS Yeast Res. 20:foaa038. doi: 10.1093/femsyr/foaa038, PMID: 32614407 PMC7334043

[ref42] Wu S. Hu C. Jin G. Zhao X. Zhao Z. K. (2010). Phosphate-limitation mediated lipid production by *Rhodosporidium toruloides*. Bioresour. Technol. 101, 6124–6129. doi: 10.1016/j.biortech.2010.02.111, PMID: 20307977

[ref43] Xiong Q. Song N. Li P. Fischer S. Konertz R. Wagle P.. (2021). RNAseq and quantitative proteomic analysis of Dictyostelium knock-out cells lacking the core autophagy proteins ATG9 and/or ATG16. BMC Genomics 22:444. doi: 10.1186/s12864-021-07756-2, PMID: 34126926 PMC8204557

[ref44] Xu J. Zhao X. Wang W. Du W. Liu D. (2012). Microbial conversion of biodiesel byproduct glycerol to triacylglycerols by oleaginous yeast *Rhodosporidium toruloides* and the individual effect of some impurities on lipid production. Biochem. Eng. J. 65, 30–36. doi: 10.1016/j.bej.2012.04.003

[ref45] Yang Z. Geng J. Yen W. L. Wang K. Klionsky D. J. (2010). Positive or negative roles of different cyclin-dependent kinase Pho85-cyclin complexes orchestrate induction of autophagy in *Saccharomyces cerevisiae*. Mol. Cell 38, 250–264. doi: 10.1016/j.molcel.2010.02.033, PMID: 20417603 PMC2861662

[ref46] Yi C. Ma M. Ran L. Zheng J. Tong J. Zhu J.. (2012). Function and molecular mechanism of acetylation in autophagy regulation. Science 336, 474–477. doi: 10.1126/science.1216990 22539722

[ref47] Yokota H. Gomi K. Shintani T. (2017). Induction of autophagy by phosphate starvation in an Atg11-dependent manner in *Saccharomyces cerevisiae*. Biochem. Biophys. Res. Commun. 483, 522–527. doi: 10.1016/j.bbrc.2016.12.112, PMID: 28013049

[ref48] Yorimitsu T. Zaman S. Broach J. R. Klionsky D. J. (2007). Protein kinase a and Sch9 cooperatively regulate induction of autophagy in *Saccharomyces cerevisiae*. Mol. Biol. Cell 18, 4180–4189. doi: 10.1091/mbc.e07-05-0485, PMID: 17699586 PMC1995722

[ref49] Yuan L. Zhang M. Yan X. Bian Y. Zhen S. Yan Y. (2016). Dynamic phosphoproteome analysis of seedling leaves in *Brachypodium distachyon* L. reveals central phosphorylated proteins involved in the drought stress response. Sci. Rep. 6:35280. doi: 10.1038/srep35280, PMID: 27748408 PMC5066223

[ref50] Zhang Y. Wolf-Yadlin A. Ross P. L. Pappin D. J. Rush J. Lauffenburger D. A.. (2005). Time-resolved mass spectrometry of tyrosine phosphorylation sites in the epidermal growth factor receptor signaling network reveals dynamic modules. Mol. Cell. Proteomics 4, 1240–1250. doi: 10.1074/mcp.M500089-MCP200, PMID: 15951569

[ref51] Zhou H. Watts J. D. Aebersold R. (2001). A systematic approach to the analysis of protein phosphorylation. Nat. Biotechnol. 19, 375–378. doi: 10.1038/86777, PMID: 11283598

[ref52] Zhou H. Ye M. Dong J. Corradini E. Cristobal A. Heck A.. (2013). Robust phosphoproteome enrichment using monodisperse microsphere-based immobilized titanium (IV) ion affinity chromatography. Nat. Protoc. 8, 461–480. doi: 10.1038/nprot.2013.010, PMID: 23391890

[ref53] Zhu Z. Zhang S. Liu H. Shen H. Lin X. Yang F.. (2012). A multi-omic map of the lipid-producing yeast *Rhodosporidium toruloides*. Nat. Commun. 3:1112. doi: 10.1038/ncomms2112, PMID: 23047670 PMC3493640

