# Supplementary material for: Regulation of autophagy and lipid accumulation under phosphate limitation in Rhodotorula toruloides
Source: Front Microbiol. 2023 Jan 26;13:1046114. doi: 10.3389/fmicb.2022.1046114 (PMC9908577; doi:10.3389/fmicb.2022.1046114)
Supplement: Supplementary file 1 [file Image_1.pdf]

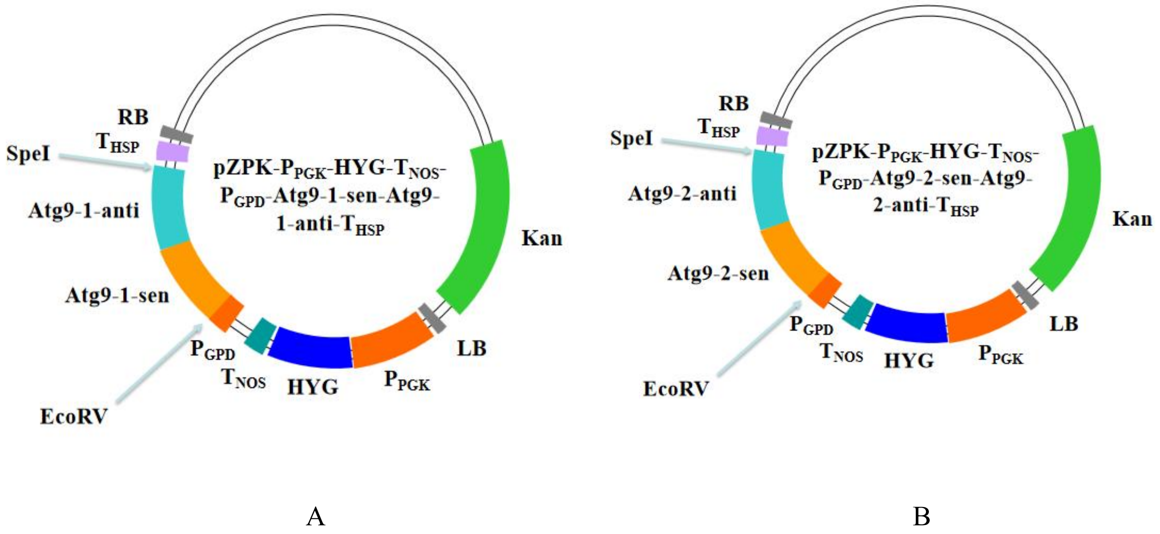

Fig. S1 Organization of the RNAi vector plasmids. P_{PGK}, the phosphoglycerate kinase promoter; HYG, hygromycin gene; T_{NOS}, the nos terminator; P_{GPD}, the glycerol 3 phosphate dehydrogenase promoter; Atg9, autophagy related protein 9; T_{HSP}, heat shock protein terminator; LB: left border; RB: right border.
