# Supplementary material for: Regulation of autophagy and lipid accumulation under phosphate limitation in Rhodotorula toruloides
Source: Front Microbiol. 2023 Jan 26;13:1046114. doi: 10.3389/fmicb.2022.1046114 (PMC9908577; doi:10.3389/fmicb.2022.1046114)
Supplement: Supplementary file 2 [file Image_2.pdf]

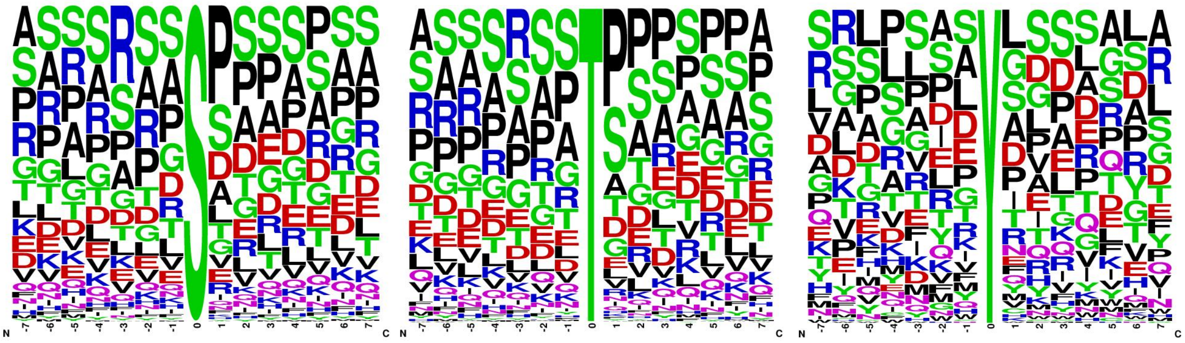

A B C

Fig. S2 Distribution of amino acids surrounding the phosphorylation site by WebLog.(A) Ser; (B) Thr; (C)Tyr.
