# Supplementary material for: Regulation of autophagy and lipid accumulation under phosphate limitation in Rhodotorula toruloides
Source: Front Microbiol. 2023 Jan 26;13:1046114. doi: 10.3389/fmicb.2022.1046114 (PMC9908577; doi:10.3389/fmicb.2022.1046114)
Supplement: Supplementary file 3 [file Image_3.pdf]

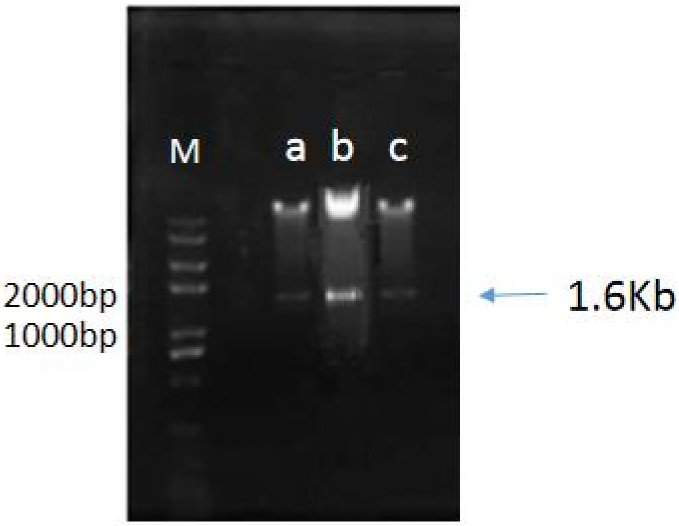

Fig. S3 The electrophoresis map for identification of recombinant pZPK-P_{PGK}-HYG-T_{NOS}-P_{GPD}-Atg9-1-sen-Atg9-1-anti-T_{HSP} (a and c) and pZPK-P_{PGK}-HYG-T_{NOS}-P_{GPD}-Atg9-2-sen-Atg9-2-anti-T_{HSP} (b) digested with EcoRV and SpeI.
