# Supplementary material for: Regulation of autophagy and lipid accumulation under phosphate limitation in Rhodotorula toruloides
Source: Front Microbiol. 2023 Jan 26;13:1046114. doi: 10.3389/fmicb.2022.1046114 (PMC9908577; doi:10.3389/fmicb.2022.1046114)
Supplement: Supplementary file 4 [file Table_1.doc]

**Table S1. Primers used in this study.**

| Primer | Sequence |
| --- | --- |
| RtAtg9-1-sen-F | CGGgatatcCGCTCTTCCCAGCTACATCAGTCTA |
| RtAtg9-1-sen-R-NcoI | CATGccatggAAGGGTCAGCACCTGCACAAAAGGC |
| RtAtg9-1-anti-F | CCactagtCGCTCTTCCCAGCTACATCAGTCTA |
| RtAtg9-1-anti-R-NcoI | CATGccatggTTCCTCAACCTCGCCTACGCCAATC |
| RtAtg9-2-sen-F | CGGgatatcAGAAGACTTGCACGACATCGCCGCG |
| RtAtg9-2-sen-R-NcoI | CATGccatggAGGTCGAAAAGCCGATCACCCAGCC |
| RtAtg9-2-anti-F | CCactagtAGAAGACTTGCACGACATCGCCGCG |
| RtAtg9-2-anti-R-NcoI | CATGccatggAAGAGGTTCAGCGTCCGCTCGAGCC |
