# Supplementary material for: Regulation of autophagy and lipid accumulation under phosphate limitation in Rhodotorula toruloides
Source: Front Microbiol. 2023 Jan 26;13:1046114. doi: 10.3389/fmicb.2022.1046114 (PMC9908577; doi:10.3389/fmicb.2022.1046114)
Supplement: Supplementary file 8 [file Table_5.doc]

**Table S5. Analysis of phosphorylation motifs using the Motif-X algorithm**

| **#** | **Motif** ^a, b^ | **No. of occurrences** | **Literature** |
| --- | --- | --- | --- |
|  | **Serine motifs** |  |  |
| 1. | **[....R..](http://motif-x.med.harvard.edu/cgi-bin/jobres.pl?jobid=20170104-16561-12476579#....R..SP......)sP......** | 312 |  |
| 2. | **[.......](http://motif-x.med.harvard.edu/cgi-bin/jobres.pl?jobid=20170104-16561-12476579#.......SP...R..)sP...R..** | 147 |  |
| 3. | **[.......](http://motif-x.med.harvard.edu/cgi-bin/jobres.pl?jobid=20170104-16561-12476579#.......SPR.....)sPR.....** | 107 | Growth-associated histone HI |
| 4. | **[R......](http://motif-x.med.harvard.edu/cgi-bin/jobres.pl?jobid=20170104-16561-12476579#R......SP......)sP......** | 94 |  |
| 5. | **[....RR.](http://motif-x.med.harvard.edu/cgi-bin/jobres.pl?jobid=20170104-16561-12476579#....RR.S.......)s.......** | 232 | PKA kinase |
| 6. | **[.......](http://motif-x.med.harvard.edu/cgi-bin/jobres.pl?jobid=20170104-16561-12476579#.......SP......)sP......** | 699 | Proline-directed |
| 7. | **[.......S](http://motif-x.med.harvard.edu/cgi-bin/jobres.pl?jobid=20170104-16561-12476579#.......SSP.....)sP.....** | 170 |  |
| 8. | **[....R..](http://motif-x.med.harvard.edu/cgi-bin/jobres.pl?jobid=20170104-16561-12476579#....R..S.D.....)s.D.....** | 56 |  |
| 9. | **[....RS.](http://motif-x.med.harvard.edu/cgi-bin/jobres.pl?jobid=20170104-16561-12476579#....RS.S.......)s.......** | 111 |  |
| 10. | **[.......](http://motif-x.med.harvard.edu/cgi-bin/jobres.pl?jobid=20170104-16561-12476579#.......S.SP....)s.SP....** | 127 |  |
| 11. | **[.......](http://motif-x.med.harvard.edu/cgi-bin/jobres.pl?jobid=20170104-16561-12476579#.......SD.E....)sD.E....** | 151 | CK2 |
| 12. | **[.R..S..](http://motif-x.med.harvard.edu/cgi-bin/jobres.pl?jobid=20170104-16561-12476579#.R..S..S.......)s.......** | 107 |  |
| 13. | **[....R..](http://motif-x.med.harvard.edu/cgi-bin/jobres.pl?jobid=20170104-16561-12476579#....R..S.G.....)s.G.....** | 57 |  |
| 14. | **[.......](http://motif-x.med.harvard.edu/cgi-bin/jobres.pl?jobid=20170104-16561-12476579#.......S...SP..)s...SP..** | 90 |  |
| 15. | **[....RA.](http://motif-x.med.harvard.edu/cgi-bin/jobres.pl?jobid=20170104-16561-12476579#....RA.S.......)s.......** | 66 |  |
| 16. | **[......D](http://motif-x.med.harvard.edu/cgi-bin/jobres.pl?jobid=20170104-16561-12476579#......DS.D.....)s.D.....** | 57 |  |
| 17. | **[....R..](http://motif-x.med.harvard.edu/cgi-bin/jobres.pl?jobid=20170104-16561-12476579#....R..S.......)s.......** | 208 | PKA and PKC kinase |
| 18. | **[.......](http://motif-x.med.harvard.edu/cgi-bin/jobres.pl?jobid=20170104-16561-12476579#.......SD.D....)sD.D....** | 77 | CK2 |
| 19. | **[.....R.](http://motif-x.med.harvard.edu/cgi-bin/jobres.pl?jobid=20170104-16561-12476579#.....R.SS......)sS......** | 64 |  |
| 20. | **[..R..S.](http://motif-x.med.harvard.edu/cgi-bin/jobres.pl?jobid=20170104-16561-12476579#..R..S.S.......)s.......** | 65 |  |
| 21. | **[.......](http://motif-x.med.harvard.edu/cgi-bin/jobres.pl?jobid=20170104-16561-12476579#.......S...D...)s...D...** | 158 |  |
| 22. | **[....S..](http://motif-x.med.harvard.edu/cgi-bin/jobres.pl?jobid=20170104-16561-12476579#....S..S.......)s.......** | 260 |  |
| 23. | **[.......](http://motif-x.med.harvard.edu/cgi-bin/jobres.pl?jobid=20170104-16561-12476579#.......S...E...)s...E...** | 115 |  |
| 24. | **[...S...](http://motif-x.med.harvard.edu/cgi-bin/jobres.pl?jobid=20170104-16561-12476579#...S...S.......)s.......** | 179 |  |
| 25. | **[.....S.](http://motif-x.med.harvard.edu/cgi-bin/jobres.pl?jobid=20170104-16561-12476579#.....S.S.......)s.......** | 136 |  |
| 26. | **[.......](http://motif-x.med.harvard.edu/cgi-bin/jobres.pl?jobid=20170104-16561-12476579#.......S...S...)s...S...** | 111 |  |
| 27. | **[.......](http://motif-x.med.harvard.edu/cgi-bin/jobres.pl?jobid=20170104-16561-12476579#.......S..S....)s..S....** | 87 |  |
| 28. | **[.......](http://motif-x.med.harvard.edu/cgi-bin/jobres.pl?jobid=20170104-16561-12476579#.......S..P....)s..P....** | 52 |  |
| 29. | **[.......](http://motif-x.med.harvard.edu/cgi-bin/jobres.pl?jobid=20170104-16561-12476579#.......S..G....)s..G....** | 51 |  |
|  | Threonine motifs |  |  |
|  | **[....R..](http://motif-x.med.harvard.edu/cgi-bin/jobres.pl?jobid=20161202-5261-27300458#....R..TP......)tP......** | 63 |  |
|  | **[....G..](http://motif-x.med.harvard.edu/cgi-bin/jobres.pl?jobid=20161202-5261-27300458#....G..TP......)tP......** | 53 |  |
|  | **[.......](http://motif-x.med.harvard.edu/cgi-bin/jobres.pl?jobid=20161202-5261-27300458#.......TSP.....)tSP.....** | 79 |  |
|  | **[.......](http://motif-x.med.harvard.edu/cgi-bin/jobres.pl?jobid=20161202-5261-27300458#.......TP.P....)tP.P....** | 49 |  |
|  | **[.....SP](http://motif-x.med.harvard.edu/cgi-bin/jobres.pl?jobid=20161202-5261-27300458#.....SPT.......)t.......** | 51 |  |
|  | **[.....P.](http://motif-x.med.harvard.edu/cgi-bin/jobres.pl?jobid=20161202-5261-27300458#.....P.TP......)tP......** | 37 | MAP kinase |
|  | **[.......](http://motif-x.med.harvard.edu/cgi-bin/jobres.pl?jobid=20161202-5261-27300458#.......TP..S...)tP..S...** | 23 |  |
|  | **[.......](http://motif-x.med.harvard.edu/cgi-bin/jobres.pl?jobid=20161202-5261-27300458#.......T.SP....)t.SP....** | 27 |  |
|  | **[.....R.](http://motif-x.med.harvard.edu/cgi-bin/jobres.pl?jobid=20161202-5261-27300458#.....R.TS......)tS......** | 26 |  |
|  | **[....R.S](http://motif-x.med.harvard.edu/cgi-bin/jobres.pl?jobid=20161202-5261-27300458#....R.ST.......)t.......** | 21 |  |
|  | **[R..S...](http://motif-x.med.harvard.edu/cgi-bin/jobres.pl?jobid=20161202-5261-27300458#R..S...T.......)t.......** | 28 |  |
|  | **[.......](http://motif-x.med.harvard.edu/cgi-bin/jobres.pl?jobid=20161202-5261-27300458#.......TP......)tP......** | 63 | Pro-directed |
|  | **[.......](http://motif-x.med.harvard.edu/cgi-bin/jobres.pl?jobid=20161202-5261-27300458#.......TD......)tD......** | 52 |  |
|  | **[.......](http://motif-x.med.harvard.edu/cgi-bin/jobres.pl?jobid=20161202-5261-27300458#.......TS......)tS......** | 79 |  |
|  | **[.......](http://motif-x.med.harvard.edu/cgi-bin/jobres.pl?jobid=20161202-5261-27300458#.......T.S.....)t.S.....** | 57 |  |
|  | **[....S..](http://motif-x.med.harvard.edu/cgi-bin/jobres.pl?jobid=20161202-5261-27300458#....S..T.......)t.......** | 48 |  |
|  | **[.....S.](http://motif-x.med.harvard.edu/cgi-bin/jobres.pl?jobid=20161202-5261-27300458#.....S.T.......)t.......** | 37 |  |
|  | **[.......](http://motif-x.med.harvard.edu/cgi-bin/jobres.pl?jobid=20161202-5261-27300458#.......TT......)tT......** | 31 |  |

^a^ Motif parameters were set to 10^-6^significance and a number of occurrences depending on the size of each data set: 50 occurrences for single pSer, 20 for pThr, and 3 for the pTyr, respectively. Lowercase "s" and "t" represent phosphorylated Ser and Thr, respectively. ^b^ "." denotes any amino acid. Kinases are listed if described in the literature.
